# Association between the RAD51 135 G>C Polymorphism and Risk of Cancer: A Meta-Analysis of 19,068 Cases and 22,630 Controls

**DOI:** 10.1371/journal.pone.0075153

**Published:** 2013-09-09

**Authors:** Wei Wang, Jia-Lin Li, Xiao-Feng He, An-Ping Li, Yong-Lin Cai, Na Xu, Shu-Mei Sun, Bing-Yi Wu

**Affiliations:** 1 Shanxi Zhendong Pharmaceutical Co. Ltd., Changzhi, Shanxi Province, China; 2 Research Center of Clinical Medicine, Nanfang Hospital, Southern Medical University, Guangzhou, Guangdong Province, China; 3 Department of Radiology, Putuo District Central Hospital, Shanghai, PR China; 4 Information Section, Peace Hospital of Changzhi Medical College, Changzhi, Shanxi Province, China; 5 Central Laboratory, Wuzhou Red Cross Hospital, Wuzhou, Guangxi Province, China; 6 Department of Hematology, Nanfang Hospital of Southern Medical University, Guangzhou, Guangdong Province, China; 7 Department of Infection Management, Nanfang Hospital, Southern Medical University, Guangzhou, Guangdong Province, China; The University of Texas M. D. Anderson Cancer Center, United States of America

## Abstract

**Background:**

RAD51 135G>C can modify promoter activity and the penetrance of BRCA1/2 mutations, which plays vital roles in the etiology of various cancer. To date, previous published data on the association between RAD51 135G>C polymorphism and cancer risk remained controversial. Recent meta-analysis only analyzed RAD51 135G>C polymorphism with breast cancer risk, but the results were also inconsistent.

**Methods:**

A meta-analysis based on 39 case-control studies was performed to investigate the association between cancer susceptibility and RAD51 135G>C. Odds ratios (OR) with 95% confidence intervals (CIs) were used to assess the association in different inheritance models. Heterogeneity among studies was tested and sensitivity analysis was applied.

**Results:**

Overall, no significant association was found between RAD51 135G>C polymorphism and cancer susceptibility in any genetic model. In further stratified analysis, significantly elevated breast cancer risk was observed in BRCA2 mutation carriers (recessive model: OR = 4.88, 95% CI = 1.10–21.67; additive model: OR = 4.92, 95% CI = 1.11–21.83).

**Conclusions:**

This meta-analysis suggests that RAD51 variant 135C homozygote is associated with elevated breast cancer risk among BRCA2 mutation carriers. Moreover, our work also points out the importance of new studies for RAD51 135G>C association in acute myeloid leukemia, especially in Caucasians, where at least some of the covariates responsible for heterogeneity could be controlled, to obtain a more conclusive understanding about the function of the RAD51 135G>C polymorphism in cancer development.

## Introduction

Recently, there is growing evidence that radicals such as reactive oxidative stress produced during metabolic process play an important role in the DNA damage which could also be caused by UV, ionizing radiation, as well as environmental chemical agents and then initiate human cancer [1]. Moreover, mutagens in living environment can produce DNA adducts, DNA damage, and DNA strand breaks [2]. If these mutagens to DNA structures are left un-repaired, genetic changes can accumulate, which may result in cell-cycle dysregulation, autonomous growth and development of invasive mechanisms, leading to carcinoma [3]. In order to maintain the integrity of the genome, mammalian cells have developed several DNA-repair mechanisms that each deal with a specific type of DNA damage. DNA-repair genes are, like detoxification enzymes, responsible for preventing cancer by protecting the integrity of the genome and are therefore considered as cancer susceptibility genes[4], [5].The association between defective DNA-repair caused by highly penetrant mutations in DNA repair genes on the one hand, and chromosomal instability and cancer predisposition on the other, is well documented for rare familial cancer syndromes like pigmentosum (XP) and ataxia telangiectasia (A–T) [5]. In contrast to the occurrence of these rare and highly penetrant mutations, the human genome contains a large number of low-penetrant single-nucleotide polymorphisms (SNPs), which make up 90% of the naturally occurring sequence variations [6], [7]. An attack from reactive oxygen species (ROS) can result in cleavage of both DNA strands, causing DNA double-strand breaks (DSBs). Double-strand breaks (DSB) damage, causing cell death or loss of genetic material, is the most injurious lesion and responsible for cancer development.

The RAD51 gene, a homologue of recA in Escherichia coli, has been mapped to chromosome 15q15.1 in humans [8]. It spans >39 kb, contains 10 exons and encodes a 339 amino acid protein (genomic accession no: NM_133487). The RAD51 gene makes a protein also called RAD51, which is essential for the repair of damaged DNA. The protein made by the BRCA2 gene binds to and regulates the RAD51 protein to fix breaks in DNA [9]. These breaks can be caused by natural or medical radiation. They also occur when chromosomes exchange genetic material (when pieces of chromosomes trade places) in preparation for cell division. The BRCA2 protein transports the RAD51 protein to sites of DNA dam age in the cell nucleus. RAD51 then binds to the damaged DNA and encases it in a protein sheath, which is an essential first step in the repair process. In addition to its association with BRCA2, the RAD51 protein also interacts with the protein made by the BRCA1 gene. By repairing DNA, these three proteins play a role in maintaining the stability of the human genome. Changes in RAD51 biosynthesis are usually preceded by changes in its gene tran scrip tion and mRNA level. Gene variability could contribute to the level of the RAD51 biosynthesis. A single nucleotide polymorphism in the 5′-untranslated region (5′-UTR) of RAD51 (a G to C substitution at position 135, the G/C polymorphism) can influence cancer risk among BRCA1/BRCA2 mutation carriers [10], [11]. In view of the potential significant role of RAD51 for tumor development, it is important to know, whether this polymorphism can account for the development and/or progression of cancer.

To date, a number of molecular epidemiological studies have been done to evaluate the association between RAD51 135G>C polymorphism and different types of cancer risk in diverse populations [12]–[64]. However, the results were inconsistent or even contradictory. Some recent meta-analysis only analyzed RAD51 135G>C polymorphism with breast cancer risk [65]–[69], but the results were also inconsistent. Gao et al. [65] found that the CC genotype was associated with a significantly increased risk of breast cancer when compared with the GG, CG, and CG/GG genotypes. Subgroup analyses showed that individuals carrying the CC genotype were associated with an elevated tumor risk in European populations and in sporadic breast cancer. Wang et al. [66] observed an overall significant increased breast cancer risk (for the recessive model CC vs. GG/CG: OR = 1.35, 95% CI = 1.05–1.74, P (heterogeneity) = 0.06). Yu et al. [67] found that there was no evidence for a significant association between 135G>C and breast cancer risk in non-BRCA1/2 mutation. The study of Sun et al. [68] had 17 studies, with significantly decreased breast cancer risk being observed in the additive model (OR = 0.995, 95% CI = 0.991–0.998) and recessive model (OR = 0.994, 95% CI = 0.991–0.998). Zhou et al. [69] suggested that RAD51 variant 135C homozygote was associated with elevated breast cancer risk among BRCA2 mutation carriers. Since then, additional several studies with a large sample size about RAD51 135G>C polymorphism with cancer risk have not been reported. Therefore, we performed a comprehensive meta-analysis by including the most recent and relevant articles to identify statistical evidence of the association between RAD51 135G>C polymorphism and risk of all cancers that have been investigated.

## Materials and Methods

### Identification and eligibility of relevant studies

A comprehensive literature search was performed using the PubMed database for relevant articles published (the last search update was July 5, 2012) with the following key words “RAD51,” “polymorphism,” and “Cancer” or “Carcinoma.” The search was limited to human studies. In addition, studies were identified by a manual search of the reference lists of reviews and retrieved studies. We included all the case–control studies and cohort studies that investigated the association between RAD51 135G>C polymorphism and cancer risk with genotyping data. All eligible studies were retrieved, and their bibliographies were checked for other relevant publications. When the same sample was used in several publications, only the most complete study was included following careful examination.

### Inclusion criteria

All human-associated studies were included if they met the following criteria: (1) only the case–control studies or cohort studies were considered; (2) evaluated the RAD51 135G>C polymorphism and the risk of cancer; (3) the genotype distribution of the polymorphism in cases and controls were described in details and the results were expressed as odds ratio (OR) and corresponding 95% confidence interval (95% CI). Major reasons for exclusion of studies were as follows: (1) not for cancer research; (2) only case population; (3) duplicate of previous publication;and (4) the distribution of genotypes among controls are not in Hardy–Weinberg equilibrium (*P*<0.01).

### Data extraction

Information was carefully extracted from all eligible studies independently by two investigators according to the inclusion criteria listed above. The following data were collected from each study: first author's name, year of publication, country of origin, ethnicity, source of controls (population-based controls and hospital-based controls), genotyping method, sample size, and numbers of cases and controls in the RAD51 135G>C genotypes whenever possible. Ethnicity was categorized as “Caucasian”, “Asian”, and “African”. When one study did not state which ethnic groups was included or if it was impossible to separate participants according to phenotype, the sample was termed as “mixed population.” Meanwhile, studies investigating more than one kind of cancer were counted as individual data set only in subgroup analyses by cancer type. We did not define any minimum number of patients to include in this meta-analysis. Articles that reported different ethnic groups and different countries or locations, we considered them different study samples for each category cited above.

### Statistical analysis

Crude odds ratios (ORs) together with their corresponding 95% CIs were used to assess the strength of association between the RAD51 135 G>C polymorphism and the risk of cancer. Following published recommendations for quality assessment in meta-analyses of genetic associations, we examined: choice of genetic models (we adopted three genetic models, avoiding assuming only one “wrong” genetic model). The pooled ORs were performed for dominant model (GC+CC *versus* GG), recessive model (GG+GC *versus* CC), additive model (GG *versus* CC), respectively. Between-study heterogeneity was assessed by calculating *Q*-statistic (Heterogeneity was considered statistically significant if *P*<0.10) [70] and quantified using the *I* ^2^ value, a value that describes the percentage of variation across studies that are due to heterogeneity rather than chance, where *I* ^2^ = 0% indicates no observed heterogeneity, with 25% regarded as low, 50% as moderate, and 75% as high [71]. If results were not heterogeneous, the pooled ORs were calculated by the fixed-effect model (we used the *Q*-statistic, which represents the magnitude of heterogeneity between-studies) [72]. Otherwise, a random-effect model was used (when the heterogeneity between-studies were significant) [73]. In addition to the comparison among all subjects, we also performed stratification analyses by cancer type (if one cancer type contained less than three individual studies, it was combined into the “other cancers” group), ethnicity, BRCA1/2 mutation status, and source of controls. Lung, bladder, esophageal, head and neck, and pancreatic cancers were defined as smoking-related cancers because tobacco smoking is an established risk factor for these cancers [74], [75]–[77]. In addition, given the roles of estrogens in the etiology of breast, cervical and ovarian cancers, these cancers were defined as estrogen-related [78], [79]. We examined whether the RAD51 135G>C polymorphism was associated with the risk of these cancers as a group as well. Moreover, sensitivity analysis was performed, including studies whose allele frequencies in controls exhibited significant deviation from the Hardy–Weinberg equilibrium (HWE), given that the deviation may denote bias. In addition, we also performed by excluding a single study each time. Last, we also ranked studies according to sample size, and then repeated this meta-analysis. HWE was calculated by using the goodness-of-fit test, and deviation was considered when *P*<0.01. Begg's funnel plots [80] and Egger's linear regression test [81] were used to assess publication bias. All of the calculations were performed using STATA version 10.0 (STATA Corporation, College Station, TX).

## Results

### Eligible studies and meta-analysis databases

**Figure 1:**
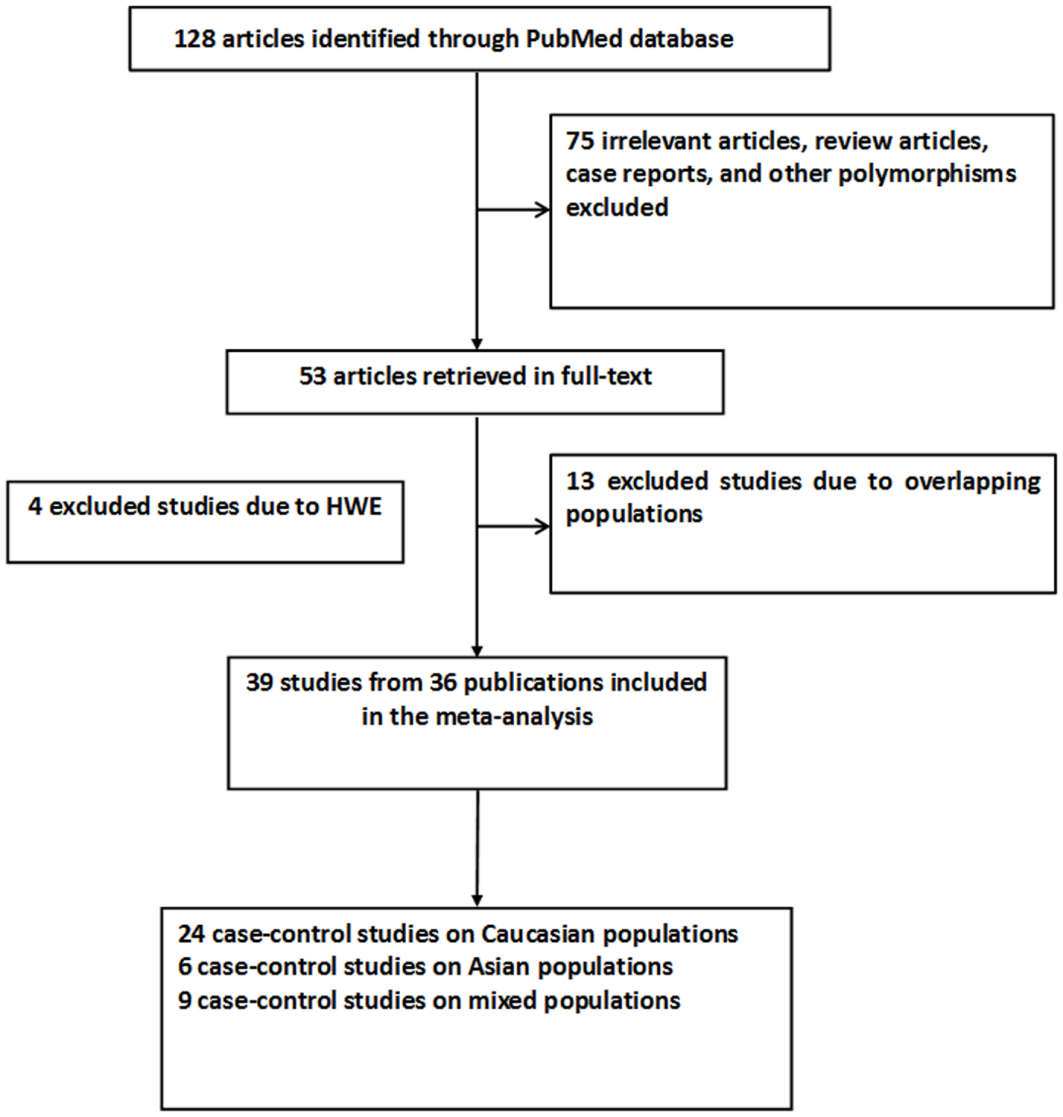
Study flow chart explaining the selection of the 39 eligible case–control studies included in the meta-analysis.

Figure 1 graphically illustrates the trial flow chart. A total of 128 articles regarding RAD51 135 G>C polymorphism with respect to cancer were identified. After screening the titles and abstracts, 75 articles were excluded because they were review articles, case reports, other polymorphisms of RAD51, or irrelevant to the current study. In addition, genotype distributions in the controls of all the eligible studies were in agreement with HWE except for four studies [45], [50], [58], [64]. Last, of these studies, 13 publications [12], [13], [15], [22], [27], [28], [30], [33], [34], [41], [53], [55], [59] were excluded because of their populations overlapped with another six included studies [20], [29], [39], [42], [50], [62]. The study of Webb et al. [17] including different case–control groups were considered as four separate studies each. Hence, as summarized in Table 1, 36 publications including 39 studies were selected among the meta-analysis, including 19,068 cases and 22,630 controls. Among the 39 studies, five studies were included in the dominant model only because they provided the genotypes of GC+CC *versus* GG as a whole. Of these, there were 20 hospital-based studies and 10 population-based studies. There were 14 breast cancer studies, 7 acute myeloid leukemia studies, 6 ovarian cancer studies, and 12 studies with the “other cancers”. Twenty-four of 39 studies were conducted in Caucasians and six studies were conducted in Asians. The remained nine studies were populations with mixed ethnicity. In addition, there were 21 estrogen-related cancers studies and 3 smoking-related cancers studies. All of the cases were pathologically confirmed.

**Table 1:** Main characteristics of all studies included in the meta-analysis.

First author/year	Country	Ethnicity	Cancer Type	Case–control	SC	GM	Genotype distribution (case/control)			HWE
							GG	GC	CC	
Levy-Lahad [25] 2001	Israel	Caucasian	Ovarian	42–90	HB	PCR	38/85	4/5		NA
Wang [26] 2001	Multiple	Mixed	Ovarian	44–263	PB	PCR-RFLP	42/238	2/25		NA
Seedhouse [14] 2004	UK	Caucasian	AML	206−186	NR	PCR-RFLP	171/166	32/18	3/2	0.171
Wang [16] 2004	USA	Caucasian	Glioma	309–342	HB	PCR-RFLP	265/301	40/41	4/0	0.840
Webb [17] 2005	Australia	Mixed	Breast	149−128	PB	PCR-RFLP	121/101	24/27	4/0	0.757
Webb [17] 2005	Australia	Caucasian	Breast	1,295−650	PB	PCR-RFLP	1100/575	188/77	7/8	0.021
Webb [17] 2005	Australia	Mixed	Ovarian	95–173	PB	PCR-RFLP	74/141	20/32	1/0	0.765
Webb [17] 2005	Australia	Caucasian	Ovarian	448–953	PB	PCR-RFLP	383/830	65/113	3/10	0.028
Auranen [18] 2005	Multiple	Caucasian	Ovarian	1,629–2,805	PB	TaqMan	1419/2440	201/355	9/10	0.746
Lee [19] 2005	Korea	Asian	Breast	782−587	HB	PCR	611/450	143/123	28/14	0.287
Sliwinski [20] 2005	Poland	Caucasian	Breast	150–150	NR	PCR-RFLP	108/106	38/41	4/3	0.912
Dufloth [21] 2005	Brazil	SA	Breast	169−119	HB	PCR-RFLP	144/103	24/13	1/3	0.026
Tarasov [23] 2006	Russia	Caucasian	Breast	151–191	NR	PCR-RFLP	111/148	36/41	4/2	0.903
Chang [24] 2006	China	Asian	Breast	189–421	HB	PCR	116/284	73/137		NA
Poplawski [31] 2006	Poland	Caucasian	Gastric	18–20	HB	PCR-RFLP	8/14	10/5	0/1	0.935
Rollinson [32] 2007	UK	Caucasian	AML	466–936	NR	TaqMan	431/817	34/115	1/4	1.000
Costa [35] 2007	Portugal	Caucasian	Breast	365–435	HB	PCR-RFLP	216/381	45/53	4/1	0.845
Lu [36] 2007	USA	Caucasian	HNSCC	716–719	HB	PCR-RFLP	624/622	91/96	1/1	0.393
Jakubowska [37] 2007	Poland	Caucasian	Ovarian	127–127	PB	PCR-RFLP	104/89	23/38		NA
Figueroa [38] 2007	Spanish	Caucasian	Bladder	1,085−1,032	HB	TaqMan	932/909	147/116	6/7	0.322
Voso [40] 2007	Italy	Caucasian	AML	160–161	NR	PCR-RFLP	125/142	33/18	2/1	0.968
Antoniou [42] 2007	Multiple	Mixed	Breast	4,443−4,069	NR	RT-PCR	3838/3485	567/565	38/17	0.747
Pharoah [29] 2007	Multiple	Caucasian	Breast	2,160–2,266	PB	TaqMan	1911/1995	236/257	13/14	0.199
Bhatla [43] 2008	USA	Mixed	AML	452–646	PB	PCR	374/555	73/85	5/6	0.418
Brooks [49] 2008	USA	Mixed	Breast	611–611	N	PCR-RFLP	516/513	88/88	7/10	0.031
Werbrouck [51] 2008	Belgium	Caucasian	HNSCC	152–157	HB	PCR-RFLP	136/134	15/23	1/0	0.848
Hu [54] 2008	China	Asian	Breast	71–85	NR	PCR-RFLP	51/59	18/23	2/3	0.930
Jakubowska [46] 2009	Poland	Caucasian	Breast	1,007–1,069	PB	PCR-RFLP	785/822	207/232	15/15	0.959
Zhang [56] 2009	China	Asian	AML	166–458	NR	PCR-RFLP	117/315	47/123	2/20	0.214
Wiśniewska-Jarosińska [58] 2009	Poland	Caucasian	Colorectal	100–236	HB	PCR-RFLP	61/169	36/44	3/23	<0.001
Palanca [48] 2010	Spain	Caucasian	BC and OC	182–208	HB	PCR	155/175	27/33		NA
Sliwinski [64] 2010	Poland	Caucasian	HNSCC	288–353	HB	PCR-RFLP	138/258	145/64	5/32	<0.001
Jara [62] 2010	Chile	SA	Breast	267–500	HB	PCR	232/441	33/58	2/1	0.835
Krupa [63] 2011	Poland	Caucasian	Endometrial	30–30	HB	PCR-RFLP	6/19	8/9	16/2	0.808
Krupa [61] 2011	Poland	Caucasian	Colorectal	100–100	HB	PCR-RFLP	61/36	36/35	3/29	0.012
Dhillon [60] 2011	Australia	Caucasian	Prostate	116–132	HB	PCR-RFLP	97/119	18/13	1/0	0.929
Liu [57] 2011	China	Asian	AML	105–704	HB	PCR-RFLP	72/511	25/175	8/18	0.809
Romanowicz-Makowska[50] 2011	Poland	Caucasian	Breast	700–708	NR	PCR-RFLP	130/178	74/396	496/134	0.005
Hamdy [47] 2011	Egypt	African	AML	50−30	HB	PCR-RFLP	39/26	9/3	2/1	0.184
Pasaje [44] 2011	Korea	Asian	Liver	285–727	HB	PCR	237/569	42/150	6/8	0.864
Smolarz [39] 2011	Poland	Caucasian	Endometrial	240–240	HB	PCR-RFLP	25/65	30/138	185/37	0.037
Gil [52] 2012	Poland	Caucasian	Colorectal	133−100	HB	PCR-RFLP	100/73	29/27	4/0	0.675
Sobti [45] 2012	India	Asian	Bladder	270−252	HB	PCR-RFLP	159/134	82/81	29/37	<0.001

HNSCC head and neck squamous cell carcinoma, NR not reported, AML acute myeloid leukemia, SA South American, N nested case–control study, HWE Hardy–Weinberg equilibrium, HB hospital-based study, PB population-based study, SC source of control, GM Genotype method.

### Quantitative synthesis

There was a wide variation in the C-allele frequency of the RAD51 135G>C polymorphism among the controls across different ethnicities. For Asian populations, the C-allele frequency was 14.06% (95% CI = 11.46%–18.18%), which was significantly higher than that in Caucasians (8.34%, 95% CI = 7.33%–18.04%, *P*<0.001). The evaluations of the association of RAD51 135G>C polymorphism with cancer risk are shown in Table 2. Overall, no significant association was found between RAD51 135G>C polymorphism and cancer susceptibility in any genetic model (dominant model: OR = 1.06, 95% CI = 0.96–1.08, *P* value of heterogeneity test [*P*_h_]<0.001, *I* ^2^ = 61.4%; recessive model: OR = 1.35, 95% CI = 0.89–2.03, *P*_h_<0.001, *I* ^2^ = 80.8%; additive model: OR = 1.46, 95% CI = 0.94–2.27, *P*_h_<0.001, *I* ^2^ = 72.8%). However, there was significant heterogeneity between studies. Hence, we then performed subgroup analysis by cancer type, smoking-related cancer, and estrogen-related cancer, there was still no significant association detected in all genetic models. We further examined the association of the RAD51 135G>C polymorphism and cancer risk according to cancer type and ethnicity (Table 3) because there was significant heterogeneity between studies. There was still no significant association detected in any ethnicity. Next, the effect of RAD51 135G>C polymorphism was evaluated in subgroup analysis according to BRCA1/2 mutation status and breast cancer (Table 4. A significant association was found only among BRCA2 mutation carriers (recessive model: OR = 4.88, 95% CI = 1.10–21.67; additive model: OR = 4.92, 95% CI = 1.11–21.83).

**Table 2:** Stratified analysis of RAD51 135G>C polymorphism on cancer risk.^1^

Variables	No. comparisons (SZ case/control)	Dominant model		Recessive model		Additive model	
		OR (95% CI)	*P*_h_/*I* 2	OR (95% CI)	*P*_h_/*I* 2	OR (95% CI)	*P*_h_/*I* 2
Overall	39 (19,068/22,630)	1.06 (0.96–1.08)	<0.001/61.4%	1.35 (0.89–2.03)	<0.001/80.7%	1.46 (0.94–2.27)	<0.001/72.8%
Cancer type							
AML	7 (1,605/3,121)	1.17 (0.84–1.65)	0.003/70.2%	1.12 (0.67–1.88)	0.123/40.2%	1.14 (0.68–1.92)	0.125/39.9%
Breast cancer	14 (11,709/11,291)	1.00 (0.93–1.07)	0.521/0.0%	1.27 (0.98–1.67)	0.198/24.3%	1.26 (0.97–1.65)	0.215/22.6%
Ovarian cancer	6 (2,388/4,411)	1.00 (0.86–1.15)	0.140/39.9%	1.23 (0.62–2.47)	0.348/5.3%	1.25 (0.62–2.49)	0.359/2.4%
Other cancer	12 (3,366/3,807)	2	<0.001/79.8%	2	<0.001/90.0%	2	<0.001/87.3%
Smoking-related	3 (1,953/1,908)	1.06 (0.88–1.27)	0.203/37.3%	0.97 (0.37–2.50)	0.738/0.0%	0.98 (0.38–2.54)	0.765/0.0%
estrogen-related	21 (14,279/15,910)	0.99 (0.93–1.06)	0.429/2.3%	1.27 (0.99–1.63)	0.265/16.5%	1.26 (0.98–1.62)	0.287/14.5%

1 All summary ORs were calculated using fixed-effects models. In the case of significant heterogeneity (indicated by *), ORs were calculated using random-effects models.

2 The results were excluded due to high heterogeneity. The bold values indicate that the results are statistically significant.

**Table 3:** Summary ORs (95% CI) and value of value of the heterogeneity of RAD51 135G>C polymorphism for studies according to ethnicity and cancer type.^1^

Ethnicity	Cancer type	No. comparisons (SZ case/control)	Dominant model		Recessive model		Additive model	
			OR (95% CI)	*P*_h_/*I* 2	OR (95% CI)	*P*_h_/*I* 2	OR (95% CI)	*P*_h_/*I* 2
Caucasian	AML	3 (832/1283)	2	<0.001/88.2%	1.08 (0.34–3.35)	0.672/0.0%	1.11 (0.36–3.44)	0.606/0.0%
	Breast cancer	6 (5028/4771)	1.04 (0.93–1.16)	0.195/32.1%	1.06 (0.70–1.60)	0.246/25.1%	1.06 (0.70–1.60)	0.243/25.5%
	Ovarian cancer	4 (2249/3975)	1.04 (0.88–1.21)	0.308/3.8%	0.77 (0.50–1.18)	0.133/46.4%	1.13 (0.55–2.34)	0.281/14.1%
Asian	Breast cancer	3 (1042/1093)	0.92 (0.72–1.16)	0.931/0.0%	1.33 (0.98–1.81)	0.785/0.0%	1.37 (0.74–2.52)	0.514/0.0%
Mixed	Breast cancer	5 (5639/5427)	0.96 (0.86–1.06)	0.924/0.0%	1.48 (0.96–2.28)	0.120/45.3%	1.46 (0.95–2.26)	0.133/43.4%

1 All summary ORs were calculated using fixed-effects models. In the case of significant heterogeneity (indicated by *), ORs were calculated using random-effects models.

2 The results were excluded due to high heterogeneity. The bold values indicate that the results are statistically significant.

**Table 4:** Meta-analysis of RAD51 135G>C polymorphism and breast cancer association according to BRCA1/BRCA2 mutation.

BRCA1/2 mutation status	Sample size (case/control)	Dominant model		Recessive model		Additive model	
		OR (95% CI)	*P*_h_/*I* ^2^	OR (95% CI)	*P*_h_/*I* ^2^	OR (95% CI)	*P*_h_/*I* ^2^
BRCA1 mutation	1 (2876/2902)	0.89 (0.77–1.03)	–	1.49 (0.80–2.76)	–	1.46 (0.79–2.71)	–
BRCA2 mutation	1 (1574/1174)	1.12 (0.89–1.41)	–	**4.88 (1.10–21.67)**	–	**4.92 (1.11–21.83)**	–
Non BRCA1/BRCA2 mutation	3 (1853/1443)	1.11 (0.90–1.36)	0.996/0.0%	0.94 (0.40–2.19)	0.218/34.1%	0.95 (0.41–2.23)	0.220/33.4%
Mixed	12 (5711/6160)	0.99 (0.89–1.09)	0.564/0.0%	1.22 (0.96–1.56)	0.570/0.0%	1.15 (0.82–1.60)	0.492/0.0%

### Test of heterogeneity and sensitivity

There was significant heterogeneity among these studies for dominant model comparison (GC+CC *versus* GG: *P*_het_<0.001), recessive model comparison (GG+GC *versus* CC: *P*_het_<0.001), and additive model comparison (GG *versus* CC: *P*_het_<0.001). Then, we assessed the source of heterogeneity for dominant model comparison (GC+CC *versus* GG) by ethnicity, cancer type, and source of controls. We found that cancer type (*P* = 0.717), ethnicity (*P* = 0.724), and the source of controls (*P* = 0.832) did not contributed to substantial heterogeneity among the meta-analysis. Although the sample size for cases and controls in all eligible studies ranged from 38 to 8,512, the corresponding pooled ORs were not qualitatively altered with or without the study of small sample. Examining genotype frequencies in the controls, significant deviation from HWE was detected in the four studies [45], [50], [58], [64]. After the inclusion of the four studies [45], [50], [58], [64] significantly departing from HWE, the results of RAD51 135G>C remained practically unchanged in the overall analysis (data not shown).

### Publication bias

**Figure 2:**
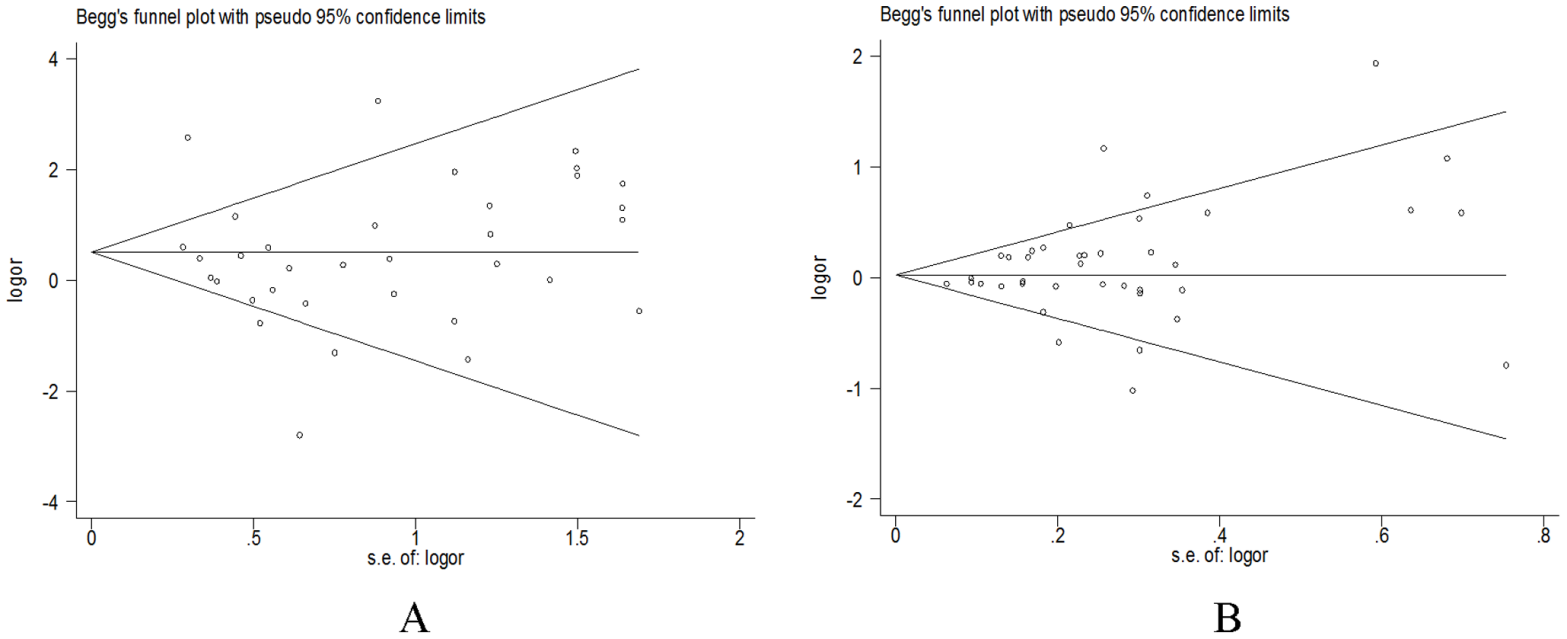
Begg's funnel plot for publication bias test in Additive model (A) and dominant model (B). Each point represents a separate study for the indicated association. Log (OR), natural logarithm of OR.

Both Begg's funnel plot and Egger's test were performed to assess the publication bias of literatures. Fig.2 lists Begg's funnel plot of allele comparison for publication bias of RAD51 135G>C (dominant model and additive model). The Egger's test results (*P* = 0.111 for dominant model, *P* = 0.120 for recessive model, and *P* = 0.525 for additive model) and Begg's funnel plot suggested no evidence of publication bias, indicating that our results were statistically robust.

## Discussion

DNA repair systems have been considered to maintain genomic integrity by countering threats posed by DNA lesions. Deficiency in the DNA repair pathways might make these lesions unrepaired or repaired incorrectly, eventually leading to genome instability or mutations which may contribute directly to cancer. Thus, genetic differences, such as single nucleotide polymorphism (SNP) may contribute to carcinogenesis [82], [83]. Previous studies have already found certain kinds of polymorphisms in DNA repair proteins are associated with cancer, such as XRCC3 (Thr241Met), OGG1 (Ser326Cys) and XPD (Lys751Gln) with breast cancer, XRCC2 (R188H G>A), and XRCC3 (T241M C>T) with ovarian cancer, XPC C/A (i11) with sporadic colorectal cancer and so on. Therefore, great interests have been aroused in the exploration of the association of SNP of DNA repair proteins and cancer risk to provide better prediction of cancer.

Homologous recombination repair (HRR), an important part of DNA repair system, is involved in the repair of double strand breaks (DSBs) [84]. Genetic polymorphisms in HRR genes, which can lead to protein haploinsufficiency have also been associated with cancer risk [85]. Double-strand break (DSB) damage, causing cell death or loss of genetic material, is the most injurious lesion and responsible for cancer development. However, it can be repaired by several DSB repair genes such as BRCA1/2 in which mutations have been proven to contribute to high risk of cancer in women [86]. RAD51 is located at chromosome position 15q15.1 [87], a region that exhibits loss of heterozygosity in a large range of cancers, including those of the lung, the colorectum, and the breast [88]. RAD51 plays a crucial role in the HRR pathway. The RAD51 135G>C polymorphism at position 135 in the 5′ UTR region may be related with RAD51 protein over-expression and DNA repair increase [89]–[91]. RAD51, a homolog of Escherichia coli RecA, is another important DSB repair gene and can interact with BRCA1 and BRCA2 proteins, functioning through homologous recombination and nonhomologous end joining [92], [93]. A number of epidemiological studies have evaluated the association between RAD51 135G>C polymorphism and cancer risk, but the results remained inconclusive. In order to resolve this conflict, this meta-analysis of 39 eligible studies including 19,068 cases and 22,630 controls was performed to derive a more precise estimation of the association between RAD51 135G>C polymorphism and risk of different types of cancer.

Overall, no significant association was found between RAD51 135G>C polymorphism and cancer susceptibility in any genetic model. In the stratified analysis by cancer type, we did not also find significant association among AML, breast cancer, and ovarian cancer. Krupa et al. [55], Jakubowska et al. [46], Chang et al. [24], Romanowicz-Makowska et al. [22], Sliwinski et al. [20], Blasiak et al. [12], Webb et al. [17], Brooks et al. [49], Kuschel et al. [27], Lee et al. [19], and Hu et al. [54] reported that the RAD51 135G>C polymorphism was not associated with the risk of breast cancer. Webb et al. [17], Dhillon [60] 2011, and Auranen et al. [18] reported that the RAD51 135G>C polymorphism was not associated with the risk of ovarian cancer. Seedhouse et al. [14], Bhatla et al. [43], and Zhang et al. [56] reported that the RAD51 135G>C polymorphism was not associated with the risk of AML. The results of our meta-analysis supported the negative association between RAD51 135G>C polymorphisms and AML, breast cancer, and ovarian cancer. In the stratified analysis by Smoking-related cancers, estrogen-related cancers, ethnicity, and BRCA1/BRCA2 mutation status, significant association was only observed between RAD51 135G>C and breast cancer risk for BRCA2 mutation carriers (Table 4; recessive model: OR = 4.88, 95% CI = 1.10–21.67; additive model: OR = 4.92, 95% CI = 1.11–21.83). As described above, the RAD51 gene product acts together with BRCA1 and BRCA2 proteins in homologous recombination and DSB repair. It is reasonable to assume that RAD51 and BRCA1/2 mutations may have interactive effects on breast cancer risk. Some previous studies presented an association of RAD51 variant allele 135C with an elevated breast cancer risk only in BRCA2 mutation carrier, but not in BRCA1 mutation carriers or non-carriers or unselected populations [15], [25], [26], [42]. In contrast, Jakubowska et al. [13], [22] observed a significantly reduced risk of breast cancer among Polish female carriers of RAD51 135C allele and BRCA1 founder mutations. Subgroup analysis on BRCA1/2 mutation status in this meta-analysis, however, confirmed the former result.

In the present meta-analysis, highly heterogeneity was observed in acute myeloid leukemia, especially in Caucasians. The reason may be acute myeloid leukemia including the hospital-based studies. The hospital-based studies have some biases because such controls may contain certain benign diseases which are prone to develop malignancy and may not be very representative of the general population. Thus, the use of a proper and representative cancer-free control subjects is very important in reducing biases in such genotype association studies. Highly heterogeneity was also observed in mix cancers, the reason may be the same polymorphisms play different roles among different cancers, because cancer is a complicated multi-genetic disease, and different genetic backgrounds may contribute to the discrepancy. Possible sources of heterogeneity, such as controls source, cancer type and ethnicity did not demonstrate the evidence of any significant variation by meta-regression. It is possible that other limitations of recruited studies may partially contribute to the observed heterogeneity. And this indicates that it may be not appropriate to use an overall estimation of the relationship between RAD51 135 G>C polymorphism and cancer risk.

Although we have put considerable efforts and resources into testing possible association between RAD51 135G>C polymorphism and cancer risk, there are still some limitations inherited from the published studies. First, our results were based on single-factor estimates without adjustment for other risk factors including alcohol usage, environmental factors and other lifestyle. At lower levels of alcohol consumption, the difference in cancer risk between the various gene carriers was less striking. And higher levels of alcohol consumption result in production of more acetaldehyde which then can exert its carcinogenic effect [94]. Second, the subgroup analysis may have had insufficient statistical power to check an association. Third, the controls were not uniformly defined. Some studies used a healthy population as the reference group, whereas others selected hospital patients without organic cancer as the reference group. Therefore, non-differential misclassification bias is possible because these studies may have included the control groups who have different risks of developing cancer of various organs. Our meta-analysis also has several strengths. First, a systematic review of the association of RAD51 135G>C polymorphism with cancer risk is statistically more powerful than any single study. Second, the quality of eligible studies included in current meta-analysis was satisfactory and met our inclusion criterion.

In conclusion, this meta-analysis suggests that RAD51 variant 135C homozygote is associated with elevated breast cancer risk among BRCA2 mutation carriers. Moreover, our work also points out the importance of new studies for RAD51 135G>C association in acute myeloid leukemia, especially in Caucasians, where at least some of the covariates responsible for heterogeneity could be controlled, to obtain a more conclusive understanding about the function of the RAD51 135G>C polymorphism in cancer development. However, it is necessary to conduct large sample studies using standardized unbiased genotyping methods, homogeneous cancer patients and well-matched controls. Moreover, further studies estimating the effect of gene–gene and gene–environment interactions may eventually lead to our better, comprehensive understanding of the association between RAD51 135G>C polymorphism and cancer risk.

## Supporting Information

Checklist S1 Prisma checklist.
(DOC)

## References

[1] Khanna KK, Jackson SP (2001) DNA double-strand breaks: signaling, repair and the cancer connection. Nat Genet 27: 247–254. 11242102 10.1038/85798

[2] Doll R, Peto R (1981) The causes of cancer: quantitative estimates of avoidable risks of cancer in the United states today. J NatlCancer Inst 66: 1191–1308. 7017215

[3] Scully C, Field JK, Tanzawa H (2000) Genetic aberrations in oral or head and neck squamous cell carcinoma (HNSCC): carcinogen metabolism, DNA repair and cell cycle control. Oral Oncol 36: 256–263. 10793327 10.1016/s1368-8375(00)00007-5

[4] Kotnisa, Sarin R, Mulherkar R (2005) Genotype, phenotype and cancer: role of low penetrance genes and environment in tumour susceptibility. J Biosci 93: 102. 10.1007/BF02705154 15824445

[5] Hu JJ, Mohrenweiser HW, Bell DA, Leadon SA, Miller MS (2002) Symposium overview: genetic polymorphisms in DNA repair and cancer risk. Toxicol Appl Pharm 185: 64–73. 10.1006/taap.2002.9518 12460738

[6] Andreassen CN, Alsner J, Overgaard J (2002) Does variability in normal tissue reactions after radiotherapy have a genetic basis–where and how to look for it? Radiother Oncol 64: 131–140. 12242122 10.1016/s0167-8140(02)00154-8

[7] Chanock S (2001) Candidate genes and single nucleotide polymorphisms (SNPs) in the study of human disease, Dis. Markers 14: 89–98. 10.1155/2001/858760 PMC3850582 11673655

[8] Shinohara A, Ogawa H, Matsuda Y, Ushio N, Ikeo K, et al (1993) Cloning of human, mouse and fission yeast recombination genes homologous to RAD51 and recA. Nat Genet 4: 239–243. 8358431 10.1038/ng0793-239

[9] Lo T, Pellegrini L, Venkitaraman AR, Blundell TL (2003) Sequence fingerprints in BRCA2 and RAD51: implications for DNA repair and cancer. DNA Repair (Amst) 18: 1015–1028. 10.1016/s1568-7864(03)00097-1 12967658

[10] Levy-Lahad E, Lahad A, Eisenberg S, Dagan E, Paperna T, et al (2001) A single nucleotide polymorphism in the RAD51 gene modifies cancer risk in BRCA2 but not BRCA1 carriers. Proc Natl Acad Sci USA 98: 3232–3236. 11248061 10.1073/pnas.051624098 PMC30636

[11] Wang WW, Spurdle AB, Kolachana P, Bove B, Modan B, et al (2001) A single nucleotide polymorphism in the 5′ untranslated region of RAD51 and risk of cancer among BRCA1/2 mutation carriers. Cancer Epidemiol Biomark Prev 10: 955–960. 11535547

[12] Blasiak J, Przybyłowska K, Czechowska A, Zadrozny M, Pertyński T, et al (2003) Analysis of the G/C polymorphism in the 5′-untranslated region of the RAD51 gene in breast cancer. Acta Biochim Pol 50: 249–253. 12673366

[13] Jakubowska A, Narod SA, Goldgar DE, Mierzejewski M, Masojć B, et al (2003) Breast cancer risk reduction associated with the RAD51 polymorphism among carriers of the BRCA1 5382insC mutation in Poland. Cancer Epidemiol Biomarkers Prev 12: 457–459. 12750242

[14] Seedhouse C, Faulkner R, Ashraf N, Das-Gupta E, Russell N (2004) Polymorphisms in genes involved in homologous recombination repair interact to increase the risk of developing acute myeloid leukemia. Clin Cancer Res 10: 2675–2680. 15102670 10.1158/1078-0432.ccr-03-0372

[15] Kadouri L, Kote-Jarai Z, Hubert A, Durocher F, Abeliovich D, et al (2004) A single-nucleotide polymorphism in the RAD51 gene modifies breast cancer risk in BRCA2 carriers, but not in BRCA1 carriers or noncarriers. Br J Cancer 90: 2002–2005. 15138485 10.1038/sj.bjc.6601837 PMC2409456

[16] Wang LE, Bondy ML, Shen H, El-Zein R, Aldape K, et al (2004) Polymorphisms of DNA repair genes and risk of glioma. Cancer Res 64: 5560–5563. 15313891 10.1158/0008-5472.CAN-03-2181

[17] Webb PM, Hopper JL, Newman B, Chen X, Kelemen L, et al (2005) Double-strand break repair gene polymorphisms and risk of breast or ovarian cancer. Cancer Epidemiol Biomarkers Prev 14: 319–323. 15734952 10.1158/1055-9965.EPI-04-0335

[18] Auranen A, Song H, Waterfall C, Dicioccio RA, Kuschel B, et al (2005) Polymorphisms in DNA repair genes and epithelial ovarian cancer risk. Int J Cancer 117: 611–618. 15924337 10.1002/ijc.21047

[19] Lee KM, Choi JY, Kang C, Kang CP, Park SK, et al (2005) Genetic polymorphisms of selected DNA repair genes, estrogen and progesterone receptor status, and breast cancer risk. Clin Cancer Res 11: 4620–4626. 15958648 10.1158/1078-0432.CCR-04-2534

[20] Sliwinski T, Krupa R, Majsterek I, Rykala J, Kolacinska A, et al (2005) Polymorphisms of the BRCA2 and RAD51 genes in breast cancer. Breast Cancer Res Treat 94: 105–109. 16261408 10.1007/s10549-005-0672-5

[21] Dufloth RM, Costa S, Schmitt F, Zeferino LC (2005) DNA repair gene polymorphisms and susceptibility to familial breast cancer in a group of patients from Campinas. Brazil. Genet Mol Res 4: 771–782. 16475125

[22] Romanowicz-Makowska H, Smolarz B, Kulig A (2005) Germline BRCA1 mutations and G/C polymorphism in the 5′-untranslated region of the RAD51 gene in Polish women with breast cancer. Pol J Pathol 56: 161–165. 16477874

[23] Tarasov VA, Aslanyan MM, Tsyrendorzhiyeva ES, Litvinov SS, Gar'kavtseva RF, et al (2006) Genetically determined subdivision of human populations with respect to the risk of breast cancer in women. Dokl Biol Sci 406: 66–69. 16572816 10.1134/s0012496606010182

[24] Chang TW, Wang SM, Guo YL, Tsai PC, Huang CJ, et al (2006) Glutathione S-transferase polymorphisms associated with risk of breast cancer in southern Taiwan. Breast 15: 754–761. 16713266 10.1016/j.breast.2006.03.008

[25] Levy-Lahad E, Lahad A, Eisenberg S, Dagan E, Paperna T, et al (2001) A single nucleotide polymorphism in the RAD51 gene modifies cancer risk in BRCA2 but not BRCA1 carriers. Proc Natl Acad Sci U S A 98: 3232–3236. 11248061 10.1073/pnas.051624098 PMC30636

[26] Wang WW, Spurdle AB, Kolachana P, Bove B, Modan B, et al (2001) A single nucleotide polymorphism in the 5′ untranslated region of RAD51 and risk of cancer among BRCA1/2 mutation carriers. Cancer Epidemiol Biomarkers Prev 10: 955–960. 11535547

[27] Kuschel B, Auranen A, McBride S, Novik KL, Antoniou A, et al (2002) Variants in DNA double-strand break repair genes and breast cancer susceptibility. Hum Mol Genet 11: 1399–1407. 12023982 10.1093/hmg/11.12.1399

[28] Pooley KA, Baynes C, Driver KE, Tyrer J, Azzato EM, et al (2008) Common single-nucleotide polymorphisms in DNA double-strand break repair genes and breast cancer risk. Cancer Epidemiol Biomarkers Prev 17: 3482–3489. 19064565 10.1158/1055-9965.EPI-08-0594

[29] Pharoah PD, Tyrer J, Dunning AM, Easton DF, Ponder BA (2007) Association between common variation in 120 candidate genes and breast cancer risk. PLoS Genet 3:e42: 401–406. 10.1371/journal.pgen.0030042 PMC1828694 17367212

[30] Romanowicz-Makowska H, Smolarz B, Zadrozny M, Kulig A (2006) Analysis of RAD51 polymorphism and BRCA1 mutations in Polish women with breast cancer. Exp Oncol 28: 156–159. 16837909

[31] Poplawski T, Arabski M, Kozirowska D, Blasinska-Morawiec M, Morawiec Z, et al (2006) DNA damage and repair in gastric cancer–a correlation with the hOGG1 and RAD51 genes polymorphisms. Mutat Res 601: 83–91. 16843501 10.1016/j.mrfmmm.2006.06.002

[32] Rollinson S, Smith AG, Allan JM, Adamson PJ, Scott K, et al (2007) RAD51 homologous recombination repair gene haplotypes and risk of acute myeloid leukaemia. Leuk Res 31: 169–74. 16890287 10.1016/j.leukres.2006.05.028

[33] Romanowicz-Makowska H, Smolarz B, Połać I, Sporny S (2012) Single nucleotide polymorphisms of RAD51G135C, XRCC2 Arg188His and XRCC3 Thr241Met homologous recombination repair genes and the risk of sporadic endometrial cancer in Polish women. J Obstet Gynaecol Res. 38(6): 918–24. 22487057 10.1111/j.1447-0756.2011.01811.x

[34] Romanowicz-Makowska H, Smolarz B, Kulig A (2006) The G/C polymorphism of RAD51 gene in breast cancer. Pol Merkur Lekarski 21: 55–58. 17007294

[35] Costa S, Pinto D, Pereira D, Rodrigues H, Cameselle-Teijeiro J, et al (2007) DNA repair polymorphisms might contribute differentially on familial and sporadic breast cancer susceptibility: a study on a Portuguese population. Breast Cancer Res Treat 103: 209–217. 17063276 10.1007/s10549-006-9364-z

[36] Lu J, Wang LE, Xiong P, Sturgis EM, Spitz MR, et al (2007) 172G>T variant in the 5′ untranslated region of DNA repair gene RAD51 reduces risk of squamous cell carcinoma of the head and neck and interacts with a P53 codon 72 variant. Carcinogenesis 28: 988–994. 17118968 10.1093/carcin/bgl225

[37] Jakubowska A, Gronwald J, Menkiszak J, Górski B, Huzarski T, et al (2007) The RAD51 135 G>C polymorphism modifies breast cancer and ovarian cancer risk in Polish BRCA1 mutation carriers. Cancer Epidemiol Biomarkers Prev 16: 270–275. 17301259 10.1158/1055-9965.EPI-06-0562

[38] Figueroa JD, Malats N, Rothman N, Real FX, Silverman D, et al (2007) Evaluation of genetic variation in the double-strand break repair pathway and bladder cancer risk. Carcinogenesis 28: 1788–1793. 17557904 10.1093/carcin/bgm132

[39] Smolarz B, Samulak D, Michalska M, Góralczyk B, Szyłło K, et al (2011) 135G>C and 172G>T polymorphism in the 5′ untranslated region of RAD51 and sporadic endometrial cancer risk in Polish women. Pol J Pathol 62: 157–162. 22102073

[40] Voso MT, Fabiani E, D'Alo' F, Guidi F, Di Ruscio A, et al (2007) Increased risk of acute myeloid leukaemia due to polymorphisms in detoxification and DNA repair enzymes. Ann Oncol 18: 1523–1528. 17761709 10.1093/annonc/mdm191

[41] Jara L, Acevedo ML, Blanco R, Castro VG, Bravo T, et al (2007) RAD51 135G>C polymorphism and risk of familial breast cancer in a South American population. Cancer Genet Cytogenet 178: 65–69. 17889711 10.1016/j.cancergencyto.2007.05.024

[42] Antoniou AC, Sinilnikova OM, Simard J, Léoné M, Dumont M, et al (2007) RAD51 135G>C modifies breast cancer risk among BRCA2 mutation carriers: results from a combined analysis of 19 studies. Am J Hum Genet 81: 1186–1200. 17999359 10.1086/522611 PMC2276351

[43] Bhatla D, Gerbing RB, Alonzo TA, Mehta PA, Deal K, et al (2008) DNA repair polymorphisms and outcome of chemotherapy for acute myelogenous leukemia: a report from the Children's Oncology Group. Leukemia 22: 265–272. 18033323 10.1038/sj.leu.2405000 PMC2914507

[44] Pasaje CF, Kim JH, Park BL, Cheong HS, Bae JS, et al (2011) Lack of association of RAD51 genetic variations with hepatitis B virus clearance and occurrence of hepatocellular carcinoma in a Korean population. J Med Virol 83: 1892–1899. 21915862 10.1002/jmv.22122

[45] Sobti RC, Kaur S, Sharma VL, Singh SK, Hosseini SA, et al (2012) Susceptibility of XPD and RAD51genetic variants to carcinoma of urinary bladder in North Indian population. DNA Cell Biol 31: 199–210. 21740187 10.1089/dna.2011.1283 PMC3272250

[46] Jakubowska A, Jaworska K, Cybulski C, Janicka A, Szymańska-Pasternak J, et al (2009) Do BRCA1 modifiers also affect the risk of breast cancer in non-carriers? Eur J Cancer 45: 837–842. 19071013 10.1016/j.ejca.2008.10.021

[47] Hamdy MS, El-Haddad AM, Bahaa El-Din NM, Makhlouf MM, Abdel-Hamid SM (2011) RAD51 and XRCC3 gene polymorphisms and the risk of developing acute myeloid leukemia. J Investig Med 59: 1124–30. 10.2310/JIM.0b013e3182281da3 21725251

[48] Palanca Suela S, Esteban Cardeñosa E, Barragán González E, de Juan Jiménez I, Chirivella González I, et al (2010) CASP8 D302H polymorphism delays the age of onset of breast cancer in BRCA1 and BRCA2 carriers. Breast Cancer Res Treat 119: 87–93. 19214744 10.1007/s10549-009-0316-2

[49] Brooks J, Shore RE, Zeleniuch-Jacquotte A, Currie D, Afanasyeva Y, et al (2008) Polymorphisms in RAD51, XRCC2, and XRCC3 are not related to breast cancer risk. Cancer Epidemiol Biomarkers Prev 17: 1016–1019. 18398049 10.1158/1055-9965.EPI-08-0065

[50] Romanowicz-Makowska H, Smolarz B, Zadrozny M, Westfal B, Baszczynski J, et al (2011) Single nucleotide polymorphisms in the homologous recombination repair genes and breast cancer risk in Polish women. Tohoku J Exp Med 224: 201–208. 21701125 10.1620/tjem.224.201

[51] Werbrouck J, De Ruyck K, Duprez F, Van Eijkeren M, Rietzschel E, et al (2008) Single-nucleotide polymorphisms in DNA double-strand break repair genes: association with head and neck cancer and interaction with tobacco use and alcohol consumption. Mutat Res 656: 74–81. 18768166 10.1016/j.mrgentox.2008.07.013

[52] Gil J, Ramsey D, Stembalska A, Karpinski P, Pesz KA, et al (2012) The C/A polymorphism in intron 11 of the XPC gene plays a crucial role in the modulation of an individual's susceptibility to sporadic colorectal cancer. Mol Biol Rep 39: 527–534. 21559836 10.1007/s11033-011-0767-5

[53] Synowiec E, Stefanska J, Morawiec Z, Blasiak J, Wozniak K (2008) Association between DNA damage, DNA repair genes variability and clinical characteristics in breast cancer patients. Mutat Res 648: 65–72. 18977234 10.1016/j.mrfmmm.2008.09.014

[54] Hu R, Wei Y, Jiang WJ, Yao WX, Long QM, et al (2008) Association of polymorphisms of N372H in BRCA2 gene and 135G/C in RAD51 gene and breast cancers. Sichuan Da Xue Xue Bao Yi Xue Ban 39: 973–975. 19253839

[55] Krupa R, Synowiec E, Pawlowska E, Morawiec Z, Sobczuk A, et al (2009) Polymorphism of the homologous recombination repair genes RAD51 and XRCC3 in breast cancer. Exp Mol Pathol 87: 32–35. 19426727 10.1016/j.yexmp.2009.04.005

[56] Zhang ZQ, Yang L, Zhang Y, Yang YH, Nie L, et al (2009) Relationship between NQO1C(609T), RAD51(G135C), XRCC3(C241T) single nucleotide polymorphisms and acute lymphoblastic leukemia. Zhongguo Shi Yan Xue Ye Xue Za Zhi 17: 523–528. 19549356

[57] Liu L, Yang L, Mi Y, Wang J, Li J, et al (2011) RAD51 and XRCC3 polymorphisms: impact on the risk and treatment outcomes of de novo inv(16) or t(16;16)/CBFβ-MYH11(+) acute myeloid leukemia. Leuk Res 35: 1020–1026. 21296419 10.1016/j.leukres.2011.01.014

[58] Wiśniewska-Jarosińska M, Sliwińfski T, Krupa R, Stec-Michalska K, Chojnacki J, et al (2009) The role of RAD 51 gene polymorphism in patients with colorectal cancer in the Polish subpopulation. Pol Merkur Lekarski 26: 455–457. 19606696

[59] Romanowicz H, Smolarz B, Baszczyński J, Zadrożny M, Kulig A (2010) Genetics polymorphism in DNA repair genes by base excision repair pathway (XRCC1) and homologous recombination (XRCC2 and RAD51) and the risk of breast carcinoma in the Polish population. Pol J Pathol 61: 206–212. 21290343

[60] Dhillon VS, Yeoh E, Fenech M (2011) DNA repair gene polymorphisms and prostate cancer risk in South Australia-results of a pilot study. Urol Oncol 29: 641–646. 19914098 10.1016/j.urolonc.2009.08.013

[61] Krupa R, Sliwinski T, Wisniewska-Jarosinska M, Chojnacki J, Wasylecka M, et al (2011) Polymorphisms in RAD51, XRCC2 and XRCC3 genes of the homologous recombination repair in colorectal cancer-a case control study. Mol Biol Rep 38: 2849–2854. 21104022 10.1007/s11033-010-0430-6 PMC3071932

[62] Jara L, Dubois K, Gaete D, de Mayo T, Ratkevicius N, et al (2010) Variants in DNA double-strand break repair genes and risk of familial breast cancer in a South American population. Breast Cancer Res Treat 122: 813–822. 20054644 10.1007/s10549-009-0709-2

[63] Krupa R, Sobczuk A, Popławski T, Wozniak K, Blasiak J (2011) DNA damage and repair in endometrial cancerin correlation with the hOGG1 and RAD51 genes polymorphism. Mol Biol Rep 38: 1163–1170. 20602259 10.1007/s11033-010-0214-z PMC3024515

[64] Sliwinski T, Walczak A, Przybylowska K, Rusin P, Pietruszewska W, et al (2010) Polymorphisms of the XRCC3 C722T and the RAD51 G135C genes and the risk of head and neck cancer in a Polish population. Exp Mol Pathol 89: 358–366. 20804747 10.1016/j.yexmp.2010.08.005

[65] Gao LB, Pan XM, Li LJ, Liang WB, Zhu Y, et al (2011) RAD51 135G/C polymorphism and breast cancer risk: a meta-analysis from 21 studies. Breast Cancer Res Treat 125: 827–835. 20640595 10.1007/s10549-010-0995-8

[66] Wang Z, Dong H, Fu Y, Ding H (2010) RAD51 135G>C polymorphism contributes to breast cancer susceptibility: a meta-analysis involving 26,444 subjects. Breast Cancer Res Treat 124: 765–769. 20396943 10.1007/s10549-010-0885-0

[67] Yu KD, Yang C, Fan L, Chen AX, Shao ZM (2010) RAD51 135G>C does not modify breast cancer risk in non-BRCA1/2 mutation carriers: evidence from a meta-analysis of 12 studies. Breast Cancer Res Treat 126: 365–371. 20461453 10.1007/s10549-010-0937-5

[68] Sun H, Bai J, Chen F, Jin Y, Yu Y, et al (2011) RAD51 G135C polymorphism is associated with breast cancer susceptibility: a meta-analysis involving 22,399 subjects. Breast Cancer Res Treat 125: 157–161. 20454923 10.1007/s10549-010-0922-z

[69] Zhou GW, Hu J, Peng XD, Li Q (2011) RAD51 135G>C polymorphism and breast cancer risk: a meta-analysis. Breast Cancer Res Treat 125: 529–535. 20623332 10.1007/s10549-010-1031-8

[70] Davey SG, Egger M (1997) Meta-analyses of randomized controlled trials. Lancet 350: 1182. 9343537 10.1016/s0140-6736(05)63833-0

[71] Higgins JP, Thompson SG, Deeks JJ, Altman DG (2003) Measuring inconsistency in meta-analysis. Br Med J 327: 557–560. 12958120 10.1136/bmj.327.7414.557 PMC192859

[72] Mantel N, Haenszel W (1959) Statistical aspects of the analysis of data from retrospective studies of disease. Natl Cancer Inst 22: 719–748. 13655060

[73] DerSimonian R, Laird N (1986) Meta-analysis in clinical trials. Control Clin Trials 7: 177–188. 3802833 10.1016/0197-2456(86)90046-2

[74] Qiu D, Kurosawa M, Lin Y, Inaba Y, Matsuba T, et al (2005) Overview of the epidemiology of pancreatic cancer focusing on the JACC Study. Epidemiol 15: S157–S67. 10.2188/jea.15.S157 PMC8639044 16127228

[75] Wei Q, Cheng L, Amos CI, Wang LE, Guo Z, et al (2000) Repair of tobacco carcinogen-induced DNA adducts and lung cancer risk: a molecular epidemiologic study. J Natl Cancer Inst 92: 1764–1772. 11058619 10.1093/jnci/92.21.1764

[76] Cohen SM, Shirai T, Steineck G (2000) Epidemiology and etiology of premalignant and malignant urothelial changes. Scand J Urol Nephrol Suppl 205:105–115. 10.1080/00365590050509869 11144890

[77] Blot WJ, McLaughlin JK, Winn DM, Austin DF, Greenberg RS, et al (1988) Smoking and drinking in relation to oral and pharyngeal cancer. Cancer Res 48: 3282–3287. 3365707

[78] Au WW, Abdou-Salama S, Sierra-Torres CH, Al-Hendy A (2007) Environmental risk factors for prevention and molecular intervention of cervical cancer. Int J Hyg Environ Health 210: 671–678. 17157560 10.1016/j.ijheh.2006.10.003

[79] Persson I (2000) Estrogens in the causation of breast, endometrial and ovarian cancers–evidence and hypotheses from epidemiological findings. J Steroid Biochem Mol Biol 74: 357–364. 11162945 10.1016/s0960-0760(00)00113-8

[80] Begg CB, Mazumdar M (1994) Operating characteristics of a rank correlation test for publication bias. Biometrics 50: 1088–1101. 7786990

[81] Egger M, Smith DG, Schneider M, Minder C (1997) Bias in meta-analysis detected by a simple, graphical test. Br Med J 315: 629–634. 9310563 10.1136/bmj.315.7109.629 PMC2127453

[82] Mendoza J, Martínez J, Hernández C, Pérez-Montiel D, Castro C, et al (2013) Association between ERCC1 and XPA expression and polymorphisms and the response to cisplatin in testicular germ cell tumours. Br J Cancer. 109(1): 68–75. 23807173 10.1038/bjc.2013.303 PMC3708571

[83] Zeljic K, Supic G, Jovic N, Kozomara R, Brankovic-Magic M, et al. (2013) Association of TLR2,TLR3, TLR4 and CD14 genes polymorphisms with oral cancer risk and survival. Oral Dis. doi:10.1111/odi.12144 10.1111/odi.12144 23796347

[84] Stark JM, Pierce AJ, Oh J, Pastink A, Jasin M (2004) Genetic steps of mammalian homologous repair with distinct mutagenic consequences. Mol Cell Biol 24: 9305–9316. 15485900 10.1128/MCB.24.21.9305-9316.2004 PMC522275

[85] Smilenov LB (2006) Tumor development: haploinsufficiency and local network assembly. Cancer Lett 240: 17–28. 16223564 10.1016/j.canlet.2005.08.015

[86] Hughes DJ (2008) Use of association studies to define genetic modifiers of breast cancer risk in BRCA1 and BRCA2 mutation carriers. Fam Cancer 7: 233–244. 18283561 10.1007/s10689-008-9181-0

[87] Takahashi E, Matsuda Y, Hori T, Yasuda N, Tsuji S, et al (1994) Chromosome mapping of the human (RECA) and mouse (Reca) homologs of the yeast RAD51 And Escherichia coli recA genes to human (15q15.1) and mouse (2F1) chromosomes by direct R-banding fluorescence insitu hybridization. Genomics 19(2): 376–8. 8188269 10.1006/geno.1994.1074

[88] Wick W, Petersen I, Schmutzler RK, Wolfarth B, Lenartz D, et al (1996) Evidence for a novel tumor suppressor gene on chromosome 15 associated with progression to a metastatic stage in breast cancer. Oncogene 12(5): 973–978. 8649814

[89] Vispe S, Cazaux C, Lesca C, Defais M (1998) Overexpression of Rad51 protein stimulates homologous recombination and increases resistance of mammalian cells to ionizing radiation. Nucleic Acids Res 26: 2859–2864. 9611228 10.1093/nar/26.12.2859 PMC147643

[90] Kim PM, Allen C, Wagener BM, Shen Z, Nickoloff JA (2001) Overexpression of human RAD51 and RAD52reduces double-strand break-induced homologous recombination in mammalian cells. Nucleic Acids Res 29: 4352–4360. 11691922 10.1093/nar/29.21.4352 PMC60192

[91] Richardson C, Stark JM, Ommundsen M, Jasin M (2004) Rad51 overexpression promotes alternative double-strand break repair pathways and genome instability. Oncogene 23: 546–553. 14724582 10.1038/sj.onc.1207098

[92] Baumann P, West SC (1998) Role of the human RAD51 protein in homologous recombination and double-stranded-break repair. Trends Biochem Sci 23: 247–251. 9697414 10.1016/s0968-0004(98)01232-8

[93] Thacker J (2005) The RAD51 gene family, genetic instability and cancer. Cancer Lett 219: 125–135. 15723711 10.1016/j.canlet.2004.08.018

[94] Visapaa JP, Gotte K, Benesova M, Li J, Homann N, et al (2004) Increased cancer risk in heavy drinkers with the alcohol dehydrogenase 1C*1 allele, possibly due to salivary acetaldehyde. Gut 53: 871–876. 15138216 10.1136/gut.2003.018994 PMC1774061

